# Dr. George E. Cragin

**Published:** 1915-11

**Authors:** 


**Dr. George E. Cragin**, Yale 1867, died at his home in Kenwood, Madison Co., N. Y., Sept. 8, aged 75.

